# Prevalence and correlates of depression and anxiety symptoms among out-of-school adolescent girls and young women in Tanzania: A cross-sectional study

**DOI:** 10.1371/journal.pone.0221053

**Published:** 2019-08-16

**Authors:** Evodius Kuringe, Jacqueline Materu, Daniel Nyato, Esther Majani, Flaviana Ngeni, Amani Shao, Deusdedit Mjungu, Baltazar Mtenga, Soori Nnko, Thomas Kipingili, Aminiel Mongi, Peter Nyanda, John Changalucha, Mwita Wambura

**Affiliations:** 1 Department of Sexual and Reproductive Health, National Institute for Medical Research, Mwanza, Tanzania; 2 Sauti Project, Jhpiego Tanzania—an affiliate of Johns Hopkins University, Dar es Salaam, Tanzania; 3 Pact Tanzania, Dar es Salaam, Tanzania; University of the Witwatersrand, SOUTH AFRICA

## Abstract

**Background:**

In sub-Saharan Africa, adolescent girls and young women (AGYW) who are out of school are at higher risk of depressive and anxiety disorders compared to their school attending peers. However, little is known about the prevalence and risk factors for these conditions among out-of-school AGYW. This study examines the prevalence of depression and anxiety and associated factors in a community sample of out-of-school AGYW in Tanzania.

**Methods:**

A cross-sectional analysis of baseline data from an on-going cluster randomized controlled trial in North-West Tanzania was conducted. A total of 3013 out-of-school AGYW aged 15 to 23 years from 30 clusters were included. Anxiety and depression were assessed using the Patient Health Questionnaire (PHQ-4), a tool comprising of PHQ-2 and Generalized Anxiety Disorders (GAD-2) screeners. Data were collected using Audio Computer-Assisted Self-Interview (ACASI). A random-effects logistic regression was fitted for binary outcomes and an ordinal logistic regression model with robust variance was used to adjust for clustering at the village level. Logistic regression and ordinal logistic regression were used to explore the associations between mental disorders symptoms and other factors.

**Results:**

The prevalence of depressive (PHQ-2 ≥ 3) and anxiety (GAD-2 ≥ 3) symptoms among out-of-school AGYW were 36% (95% CI 33.8%-37.3%) and 31% (95% CI 29.0%-32.3%) respectively. Further, using the PHQ-4 tool, 33% (95% CI 30.8%-34.2%) had mild, 20% (95% CI 18.3%-21.1%) moderate and 6% (95% CI 5.5%-7.2%) had severe symptoms of anxiety and depression. After adjusting for other covariates, two factors most strongly associated with having anxiety symptoms were violence experience from sexual partners (AOR = 1.63, 95% CI: 1.36–1.96) and HIV positive status (AOR = 1.54, 95% CI: 1.03–2.31). Likewise, living alone, with younger siblings or others (AOR = 2.51, 95% CI: 1.47–4.29) and violence experience from sexual partners (AOR = 1.90, 95% CI: 1.59–2.27) were strongly associated with depression symptoms. Having savings (AOR = 0.81, 95% CI: 0.70–0.95) and emotional support (AOR = 0.82, 95% CI: 0.67–0.99) were protective against depression and anxiety, respectively.

**Conclusion:**

Depressive and anxiety symptoms are prevalent among out-of-school AGYW in Tanzania. The findings emphasize the need to strengthen preventive interventions and scale-up mental health disorder screening, referral for diagnosis and management.

## Introduction

Depression and anxiety are common mental illnesses worldwide[1]. The World Health Organization (WHO) estimates that about 260 million people were living with anxiety disorders and 300 million people were suffering from depression globally in 2017 [2]. In 2016, depressive and anxiety disorders accounted for 7% of the total disability-adjusted life years (DALYs) lost among women of reproductive age(15–49 years) worldwide[3], and were the leading cause of DALY’s lost among women aged 15–29 in Africa [1]. These two conditions were among the top five global causes of DALYs lost among adolescent girls aged 15 to 19 years in 2015 [4]. Depressive and anxiety disorders are often comorbid[5].

Several factors are associated with an increased risk of depression and anxiety. A systematic review of global mental health among children and adolescents showed that risk factors can be categorized into life-long risk factors, such as genetic background or exposure to harmful substances in utero, and age-specific risk factors such as substance use, developmental-behavioral disorders among others[6]. Evidence from South Africa, Uganda and Kenya show that age-specific risk factors include poverty, lower socio-economic status, lack of social capital and support, substance use and exposure to violence and traumatic events[7–10]. Comorbid substance use and mental health disorders are also common, creating a double burden [11][12]. Some of the protective factors for mental health disorders in sub-Saharan Africa include good parenting and social support [13,14]. Often, the protective and risk factors coexist during a person’s life course thus limiting an understanding of the onset and progression of these mental disorders[6]. Globally, most mental health disorders diagnosed in adulthood have their onset during a young age, either in childhood or adolescence[15–17].

Mental health disorders, particularly among adolescent girls and young women (AGYW), are generally under-researched in sub-Saharan Africa. Although there is limited literature on the topic, one study in Malawi suggested that AGYW who are out-of-school are at a higher risk of mental health disorders compared to their school-attending peers [18]. In Malawi, the risk factors for mental health disorders among AGYW who are out of school were poverty, daily hassles and limited social capital [18]. A strong association between depression and/or anxiety disorders and HIV infection among AGYW has been described both in SSA and elsewhere[19–23]. Depression and anxiety disorders may compromise adaptive coping mechanisms and cause suboptimal decision-making capacity, as documented in African-American and Ugandan young women, leading to risky sexual risk behaviors[19–21]. Conversely, HIV infection may predispose adolescents to depression and/or anxiety through stress-induced by knowledge of HIV status, enacted or internalized stigma, blame, victimization and violence, as seen in Zimbabwe and South Africa[22,23].

One of the ways to promote mental health and inform future interventions among out-of-school AGYW is to create awareness on the burden and risks of anxiety and depression and preventive measures. The literature on depressive and anxiety disorders among out of school AGYW in Tanzania is scarce, with the majority of studies focusing on hospital-based populations[24] and women around the peripartum period [25,26]. In keeping with recent recommendations about setting programmatic agendas for mental health in SSA [5], a clear understanding of the burden, protective and risk factors is paramount [6]. This study examines the prevalence of and associated factors to depression and anxiety among out-of-school AGYW in Tanzania.

## Methods

### Study design and setting

This is a cross-sectional analysis of baseline data from an on-going cluster randomized controlled trial known as the CARE study. The study was initiated in October 2017. Baseline data for the study, from which the results are drawn, was collected from October to December 2017.

CARE study is an impact evaluation trial aiming to assess the impact of cash transfer to adolescent girls and young women to reduce sexual risk behavior in Tanzania. CARE study is a two-parallel arm cluster-randomized controlled trial implemented among AGYW aged 15–23 years and who are out-of-school in three localities in Shinyanga region, North West of Tanzania.

The CARE study is conducted in association with the Sauti program, which is a combination HIV prevention program funded by PEPFAR through USAID and conducted jointly by the Ministry of Health, Community Development, Gender, the Elderly and Children (MOHCDGEC) and led by Jhpiego, an affiliate of Johns Hopkins University. Since 2015, Sauti has provided behavioral, biomedical and structural interventions to key and vulnerable populations to prevent HIV infections and provide services or link those affected with care, treatment, and support. CARE study is conducted in Kahama town council, and Msalala and Ushetu district councils, which are areas that have been supported with interventions for AGYW since 2015.

The village (or neighborhood/*mtaa* for urban authorities) was used as the unit for randomization to prevent dilution of the intervention effect where participants belonging to intervention and control arms live in the same household and decide to share the cash provided (intervention). However, data on whether some participants came from the same household were not collected by the study. Villages or *mtaa* were eligible for inclusion in CARE study if they were within Sauti project coverage areas and had 110 to 150 AGYW according to the household survey (girls’ roster) conducted by the Sauti project. Thirty clusters were randomly assigned into intervention and control arms, matched in pairs based on locality (rural versus urban) and HIV risk (high; where there are mines, plantations and fishing areas versus low where none of these features exists).

In the CARE study, the intervention arm, AGYW receive a direct cash transfer of 70,000 Tanzania shillings (~33 USD) quarterly, through mobile phone-based money transfer over 18 months. AGYW in the control clusters do not receive cash. In both arms, AGYW receive HIV combination prevention interventions. These include biomedical interventions (such as sexually transmitted infection and gender-based violence screening and referral to care and treatment centers for antiretroviral therapy (ART)), behavioral interventions (such as social and behavior change communication (SBCC)group education sessions and HIV testing and counseling), and structural interventions (including training on positive parenting skills, entrepreneurship and employability skills, savings and loans, and literacy skills). The combination prevention interventions are offered as part of the vulnerability-tailored, context-specific services offered by the Sauti project in Tanzania.

### Sample size, eligibility criteria, and recruitment

Sauti project conducted a household survey to identify AGYW who are eligible for the cash transfer program (CTP). The survey identified AGYW aged 15–23 years who were out of school and residents of respective Sauti project coverage areas. In addition, the AGYW consented to take part in the project. Parental/guardian permission was sought for those below 18 years.

Prior to CARE study enrollment, AGYW identified through the household survey were invited to take part in the group SBCC sessions to fulfill the criteria for the CTP. During SBCC sessions, AGYW were given information about CARE study. After the completion of the sessions, SBCC facilitators informed potential participants in their groups about CARE study eligibility criteria and enrolment procedures. Participants were eligible for CARE study if they were current residents of the study villages or *mitaa*; aged from 15 to 23 years and currently out of school (has never been to school, completed primary or secondary school and did not continue with further education or dropped out of school at least one month before study enrolment as documented in the girls' roster by the household survey). Other eligibility criteria included graduating from ten hours of SBCC sessions; registered into the cash transfer program for those residing in the intervention clusters; and consented to participate in the study.

During CARE study recruitment, potential participants were informed about the location and dates of recruitment. This way, potential participants were able to reach the registration desk for pre-screening consent and eligibility screening until the sample required was reached.

Three thousand one hundred and five (3105) participants were screened for eligibility. Of these, 3071 participants met the eligibility criteria. Of the eligible potential participants, 3055 consented for study participation and 16 participants refused to take part in the study. Among participants who consented, 3014 participants completed the baseline survey.

### Data collection and procedures

Data collection was conducted by trained research assistants. The research assistants were trained on research ethics, interviewing skills, data management as well as study-specific procedures. In addition, all research assistants received online training in good clinical practice (GCP). The interviews were conducted in private settings within the community to ensure the privacy of the study participants.

At the study site, the participants were first screened for eligibility then consented for study participation. After consenting (and assent for those below 18 years), the participants completed an interview. This was followed by blood collection and pre-and post-test HIV testing and counseling following the Tanzania national HIV testing and counseling guidelines [27]. Participants with at least 18 years of age and those below 18 years but who were sexually active, pregnant, married or had children (mature minors) consented for HIV testing[27]. Those below 18 years and not mature minors assented for HIV testing and consent was obtained from parents/guardians. The interview was completed using Audio Computer-Assisted Self Interview (ACASI), with a structured questionnaire programmed on a tablet (S1 Questionnaire). ACASI is an electronic self-administered questionnaire, where participants privately listen to pre-recorded interview questions using earphones attached to a tablet. ACASI was used in order to improve privacy, confidentiality and reduce social desirability in responding to interview questions [28]. Trained female interviewers assisted the participants whenever needed.

To verify the reliability of the questions in relation to language, the questionnaire was translated into Kiswahili language and back-translated to the English language by independent translators. The Kiswahili questionnaire was then voice-recorded by a female interviewer and programmed onto tablets. A separate set of questions was also included in the questionnaire for training participants on ACASI navigation. Participants practiced on the use of the tablets in answering questions until they felt comfortable to begin the actual interview. Two questions were included in the questionnaire to ensure accurate ACASI comprehension and use of the tablet.

### Measures

Mental health screening was conducted using the 4-item Patient Health Questionnaire for depression and anxiety (the PHQ-4) developed by Kroenke and colleagues[29]. The PHQ-4 is a very short tool composed of the PHQ-2 screening tool for depression and generalized anxiety disorders (GAD) screener (GAD-2) [29]. PHQ-2 collects self-reports of two core symptoms of depression while GAD-2 collects two core symptoms of anxiety using measures taken from the Diagnostic and Statistical Manual of Mental Disorders, Fourth Edition (DSM-IV) [29]. When evaluated against Structured Clinical Interview for DSM-IV, the PHQ-2 showed a sensitivity and specificity of 87% and 78% respectively for major depressive disorder [30]. While the receiver operating curve analysis for GAD-2 showed an area under the curve (AUC) of 0.80–0.91 for the most common anxiety disorders namely; generalized anxiety disorder, panic disorder, social anxiety disorder and posttraumatic stress disorder[29]. The PHQ-4 showed the AUC of 0.835 and 0.787 for depression and anxiety respectively in a validation conducted among college students in the United States [31]. The observed Cronbach’s alpha of the PHQ-4 in our sample was 0.79, suggesting a reliable internal consistency[32,33].

The PHQ-4 tool was included in the baseline questionnaire which participants filled in using the ACASI described above. For depression, the tool assessed feelings of ‘Little interest or pleasure in doing things’ and ‘Feeling down, depressed or hopeless’ during the last two weeks. For anxiety, the tool assessed; ‘Feeling nervous or anxious or on edge’ and ‘Not being able to stop or control worrying’ during the last two weeks. Participants responded to these four points using a 4-point Likert-type of options; ‘not at all’ = 0, ‘several days’ = 1, ‘more than half the days’ = 2, ‘nearly every day’ = 3 [29]. Participants were assigned a probable diagnosis of anxiety if the score for the two core symptoms of anxiety was equal to or more than three (GAD-2 ≥ 3)[29]. A score of≥ 3 for the two core symptoms of depression resulted in a positive screen for depression (PHQ-2 ≥ 3) [29]. A probable diagnosis of anxiety and depression was reached using the composite score (PHQ-4)[29]. The overall measure of anxiety and depression was graded as normal (0–2), mild (3–5), moderate (6–8), and severe (9–12) [29].

Age was grouped into two categories: 15–19 and 20–23 years to reflect differing situations of young women in the younger or older stages of adolescence. Marital status was grouped into three categories; single, currently married (including those who were in polygamous, monogamous or cohabiting relationships) and formerly married (including those who were separated, divorced or widowed).

Literature suggests an association between family composition and mental health symptoms [34]. Participants were interviewed about who they lived with at the time of the baseline survey. The participant could indicate whether she lived alone, with husband, parent(s), relative(s), elder siblings, younger siblings or others. These seven measures were combined into a three-category family composition variable; participants living with parents, relatives or elder siblings were grouped in one group, those reporting to be living with husband in another group and those living alone, with younger siblings or others in the third group.

The socio-economic group membership was defined as being a member of a savings and loan group or other social cohesion and mutual-help group. Any AGYW who reported to have ever used heroin, cocaine, sniffing glue or cannabis was grouped as ever used illicit drugs. Sex work was defined as ever negotiated with a man to be paid some money in exchange for offering sexual intercourse in the past six months.

Violence experience from sexual partners was assessed using the World Health Organization definition and the types of violence measured were grouped as emotional, physical and sexual violence [35]. Participants who reported to be sexually active were asked if they ever experienced each of the types of violence perpetrated by any of their sexual partners in the last six months. Participants who answered affirmatively to any type of violence were categorized as experienced violence from a sexual partner in the last six months.

Two proxy measures of deprivation were collected as part of the study. Participants who reported to belong to poor households supported by Tanzania Social Action Fund (TASAF) or reported to have slept hungry due to lack of food at any time in the past month prior to the baseline survey were categorized as physically deprived. Participants who reported to have someone to rely on for emotional or psychological support were categorized as having emotional/psychological support.

HIV testing was conducted on-site following Tanzania’s national HIV testing and counseling guidelines[27], using SD Bioline HIV-1/2 3.0 (Standard Diagnostics, Inc., Korea) and UniGold Recombigen HIV test [Trinity Biotech, Bray, County Wicklow, Ireland] rapid tests. Discordant HIV test results were confirmed using BioElisa HIV 1+2 Ag/Ab Test (Biokit S.A, Barcelona, Spain). All study participants were tested for HIV regardless of previous knowledge of HIV status. Participants who tested HIV positive received escorted referral to care and treatment centers.

### Data management

ACASI data were collected using tablets and sent to the server daily via a secure file transfer protocol. The server is located at the National Institute for Medical Research (NIMR), Mwanza center. The data were extracted by the data manager for cleaning, checking for completeness and consistency. Data queries were generated and sent back to the field for resolution while the data collection team was on the site. This process continued until all the queries were resolved. Later the final analytical datasets were produced (S1 Minimal data set).

### Statistical analysis

Data were cross-sectionally analyzed using SAS 9.4 (SAS Institute Inc.; Cary, North Carolina) and STATA 14 (College Station, TX: StataCorp LP). Anxiety (GAD-2), depression (PHQ-2) and combined anxiety and depression (PHQ-4) were treated as separate outcome variables and their association with socio-demographic characteristics, sexual partner violence, sex work, and HIV status were assessed. Descriptive analysis (frequencies and proportions) of variables was done to describe the socio-demographic characteristics, the prevalence of anxiety, depression, anxiety and depression, HIV serostatus and sex work.

The random-effects logistic regression was fitted with binary outcomes to account for the random effect of each village (cluster level) [36][37]. Due to the categorical nature of depression-anxiety co-morbidity outcome, an ordinal logistic regression model with robust variance was used to adjust for clustering at the village level[37]. The robust variance estimator at the cluster level approximates a comparable Generalized Estimating Equations (GEE) ordinal model [38,39]. The anxiety (GAD-2) and depression (PHQ-2) outcomes were used with binary logistic regression separately while anxiety and depression grades (PHQ-4) outcome was used with the proportional odds model (POM) for ordinal logistic regression to assess their relationship with predictors. When the POM assumptions are met, the odds ratio for each independent variable is constant across all possible collapsing of the outcome variable [40]. In our case, the POM predicts the probability of being normal (having no symptoms of anxiety and depression) or having severe symptoms of anxiety and depression across the entire range of anxiety and depression outcomes [40]. Brant test in Stata was used to test if the parallel regression assumption has been met.

In both cases (binary and ordinal logistic regression), univariate analysis models were fitted first to look at the relationship between the response and each variable separately. Then, potential variables with likelihood ratio p-value <0.15 were selected and included in the multivariate analysis. Variables that were not significant and not confounding the effect of other variables in the multivariate model were removed. A full sample was used in the multivariate model except for variables restricted to sexually active participants (i.e. engaged in sex work and experienced violence from a sexual partner in the last six months. For both the binary and ordinal logistic regression models, the interpretation was done on the best fitting model, selected on the basis of QICU, a modified version of Quasi-likelihood under the Independence model Criterion (QIC) statistic [41]. QICu, defined as Q+2p, adds a penalty (2p) to the quasi-likelihood (Q), where p is the number of parameters in the model [41]. Similar to the Akaike Information Criterion (AIC), a model with smaller QICu is preferred [41]. In addition, variables from the selected model were tested for interaction. Unadjusted Odds Ratio (UOR) and adjusted Odds Ratio (AOR) with 95% confidence interval (95% CI) were computed and reported where appropriate.

### Ethical considerations

CARE study received ethical approval from the Medical Research Coordinating Committee of the National Institute for Medical Research (NIMR/HQ/R.8a/Vol.IX/2287) and from the Johns Hopkins University Institutional Review Board (00007976). CARE study is registered at ClinicalTrials.gov, number NCT03597243. All participants provided written informed consent prior to study enrollment. AGYW who were at least 18 years provided voluntary informed consent for study participation. While participants below 18 years assented and their parents or guardians provided informed consent for their participation in the study. A guardian was defined as any individual authorized under applicable local law to consent on behalf of a participant under 18 years of age for participation in the research study [42].

## Results

### Characteristics of study participants

A total of 3014 study participants were interviewed in CARE study, but one participant was dropped due to ACASI comprehension issues. Of the 3013 study participants, the mean age was 20 years (Standard deviation 2.5 years), with 1691 (56.1%) being 20 to 23 years old. 1603 (53.2%) of the participants had completed seven years of elementary education (primary school) and 1557 were living with parents/relatives or elder siblings (51.7%). Of the study participants, 1508 reported being currently married (50.1%), and 1812 (60.1%) had children. Among sexually active participants (n = 2276), 816 (35.6%) reported experiencing violence from sexual partners in the last six months. 387 (17.0%) participants reported negotiating to be paid some amount of money in exchange for sexual intercourse with men (sex work) in the last 6 months. And, 107 (3.6%) study participants tested positive for HIV infection (Table 1).

**Table 1:** Baseline participant characteristics by anxiety and depression symptoms.

	Total n = 3013	Anxiety (GAD-2 ≥ 3) n = 924(30.7%)	Depression (PHQ-2 ≥ 3) n = 1071(35.5%)	Anxiety and depression (PHQ-4)		
				Mild n = 979 (32.5%)	Moderate n = 594 (19.7%)	Severe n = 192 (6.4%)
**Age (Years):**						
15–19	1322 (43.9)	380 (28.7)	396 (30.0)	418 (31.6)	219 (16.6)	75 (5.7)
20–23	1691 (56.1)	544 (32.2)	675 (39.9)	561 (33.2)	375 (22.2)	117 (6.9)
**Marital status:**						
Single	1318 (43.7)	376 (28.5)	423 (32.1)	441 (33.5)	225 (17.1)	76 (5.8)
Currently-married	1508 (50.1)	488 (32.4)	582 (38.6)	478 (31.7)	333 (22.1)	100 (6.6)
Formerly-married	187 (6.2)	60 (32.1)	66 (35.3)	60 (32.1)	36 (19.3)	16 (8.6)
**Education status:**						
No formal/Incomplete primary education	850 (28.2)	251 (29.5)	281 (33.1)	272 (32.0)	153 (18.0)	55 (6.5)
Complete primary school	160 3(53.2)	476 (29.7)	548 (34.2)	497 (31.0)	306 (19.1)	93 (5.8)
Complete/incomplete secondary school	560 (18.6)	197 (35.2)	242 (43.2)	210 (37.5)	135 (24.1)	44 (7.9)
**Family composition (Lives with):**						
Parents/relative/elder siblings	1557 (51.7)	442 (28.4)	503 (32.3)	508 (32.6)	271 (17.4)	94 (6.0)
Husband	1393 (46.2)	456 (32.7)	531 (38.1)	452 (32.5)	303 (21.8)	91 (6.5)
Alone/younger siblings/others	63 (2.1)	26 (41.3)	37 (58.7)	19 (30.2)	20 (31.8)	7 (11.1)
**HIV status** ^**a**^:						
Positive	107 (3.6)	43 (40.2)	48 (44.9)	33 (30.8)	24 (22.4)	15 (14.0)
Negative	2894 (96.4)	878 (30.3)	1020 (35.3)	942 (32.6)	568 (19.6)	177 (6.1)
**Has children:**						
**Yes**	1812 (60.1)	575 (31.7)	685 (37.8)	589 (32.5)	383 (21.1)	123 (6.8)
**No**	1201 (39.9)	349 (29.1)	386 (32.1)	390 (32.5)	211 (17.6)	69 (5.8)
**Has savings:**						
**Yes**	1518 (50.4)	450 (29.6)	502 (33.1)	513 (33.8)	275 (18.1)	86 (5.7)
**No**	1495 (49.6)	474 (31.7)	569 (38.1)	466 (31.2)	319 (21.3)	106 (7.1)
**Has microbusiness:**						
Yes	1277 (42.4)	389 (30.5)	468 (36.7)	402 (31.5)	266 (20.8)	77 (6.0)
No	1736 (57.7)	535 (30.8)	603 (34.7)	577 (33.2)	328 (18.9)	115 (6.6)
**Physical deprivation:**						
Yes	2532 (84.0)	778 (30.7)	892 (35.2)	827 (32.7)	501 (19.8)	156 (6.2)
No	481 (16.0)	146 (30.4)	179 (37.2)	152 (31.6)	93 (19.3)	36 (7.5)
**Has emotional/psychological support:**						
Yes	2376 (78.9)	705 (29.7)	824 (34.7)	776 (32.7)	467 (19.7)	130 (5.5)
No	637 (21.1)	219 (34.5)	247 (38.8)	203 (31.9)	127 (19.9)	62 (9.7)
**Socio-economic group member:**						
Yes	2318 (76.9)	707 (30.5)	820 (35.4)	772 (33.3)	440 (19.0)	150 (6.5)
No	695 (23.1)	217 (31.2)	251 (36.1)	207 (29.8)	154 (22.2)	42 (6.0)
**Ever used illicit drugs:**						
Yes	202 (6.7)	63 (31.2)	61 (30.2)	62 (30.7)	36 (17.8)	12 (5.9)
No	2811 (93.3)	861 (30.6)	1010 (35.9)	917 (32.6)	558 (19.9)	180 (6.4)
**Locality:**						
Urban	1012 (33.6)	322 (31.8)	374 (37.0)	344 (34.0)	193 (19.1)	78 (7.7)
Rural	2001 (66.4)	602 (30.1)	697 (34.8)	635 (31.7)	401 (20.0)	114 (5.7)
**HIV risks:**						
High	2031 (67.4)	627 (30.9)	730 (35.9)	670 (33.0)	403 (19.8)	136 (6.7)
Low	982 (32.6)	297 (30.2)	341 (34.7)	309 (31.5)	191 (19.5)	56 (5.7)
Experienced violence from sexual partner^*^:						
Yes	816 (35.9)	324 (39.7)	397 (48.7)	274 (33.6)	232 (28.4)	85 (10.4)
No	1460 (64.2)	412 (28.2)	482 (33.1)	485 (33.2)	259 (17.7)	73 (5.0)
Engaged in sex work*^,^ ^**b**^:						
Yes	387 (17.0)	150 (38.8)	159 (41.1)	137 (35.4)	91 (23.5)	28 (7.2)
No	1889 (83.0)	586 (31.0)	720 (38.1)	622 (32.9)	400 (21.2)	130 (6.9)

^**a**^ 12 observations missing

^**b**^ Defined as explicit and prior negotiation to be paid some money in exchange for offering sexual intercourse

^*^ Relevant to sexually active participants; characteristics reported in the last six months

Overall 924 (30.7%, 95% CI: 29.0%-32.3%) of study participants screened positive for anxiety (GAD-2 ≥ 3). Of the participants living with HIV, 43 (40.2%) screened positive for anxiety. Among participants who reported to have experienced violence from sexual partners in the last six months 324 (39.7%) had anxiety symptoms. In addition, of those who reported having engaged in sex work in the last six months, 150 (38.8%) screened positive for anxiety (Table 1).

One thousand and seventy-one (35.5%, 95% CI: 33.8%-37.3%) study participants screened positive for depression (PHQ-2 ≥ 3). Of participants aged 20 to 23 years, 675 (39.9%) screened positive for depression. Among those currently married, 582 (38.6%) screened positive for depression while nearly two-thirds,37(58.7%) of the study participants who were either living alone, living with their younger siblings or others also had a positive screening for depression. Moreover, among the 107study participants living with HIV, 48 (44.9%) had symptoms of depression. While, among participants who reported experiences of violence from sexual partners in the last six months, 397 (48.7%) had a positive depression screen (Table 1).

Using PHQ-4, 979 (32.5%, 95% CI: 30.8%-34.2%)) participants had mild, 594 (19.7%, 95% CI: 18.3%-21.1%)) had moderate, and 192 (6.4%, 95% CI: 5.5%-7.2%) had severe symptoms of anxiety and depression. 375 (22.2%) study participants aged 20–23 years and 135 (24.1%) participants with either incomplete or complete secondary school education reported moderate symptoms of anxiety and depression. Further, 15 (14.0%) participants living with HIV reported severe symptoms of anxiety and depression (Table 1).

### Predictors of anxiety symptoms (GAD-2 ≥ 3)

In an unadjusted model; age, educational status, family composition, emotional support, HIV status, having experienced violence from a sexual partner and engaged in sex work in the last six months were significantly associated with reporting symptoms of anxiety among study participants (Table 2).

**Table 2:** Logistic regression analysis for anxiety and its predictors among AGYW.

	Anxiety (GAD-2 ≥ 3)	
	*p*-value,	*p*-value,
	UOR (95% CI)	AOR (95%CI)
**Age (Years)**	*p = 0*.*043*	* p = 0*.*81*
15–19	1	1
20–23	1.18 (1.01, 1.38)	1.02 (0.86, 1.22)
**Marital status**	* p = 0*.*08*	
Single	1	
Currently-married	1.20 (1.02, 1.41)	
Formerly-married	1.18 (0.85, 1.65)	
**Education status**	*p = 0*.*039*	*p = 0*.*040*
No formal/Incomplete primary education	1	1
Complete primary school	1.01 (0.84, 1.21)	1.05 (0.87, 1.26)
Complete/In complete secondary school	1.30 (1.03, 1.63)	1.36 (1.07,1.74)
**Family composition:**	*p = 0*.*008*	*p = 0*.*049*
Parents/relative or elder siblings	1	1
Husband	1.23 (1.05, 1.44)	1.23 (1.02, 1.47)
Alone/younger siblings/other	1.77 (1.06, 2.96)	1.49 (0.87, 2.53)
**Has children:**	* p = 0*.*12*	
No	1	
Yes	1.13 (0.97, 1.33)	
**Has savings:**	*p = 0*.*22*	
No	1	
Yes	0.91 (0.78, 1.06)	
**Has micro business:**	*p = 0*.*83*	
No	1	
Yes	0.98 (0.84, 1.15)	
**Physical deprivation:**	*p = 0*.*87*	
No	1	
Yes	1.02 (0.82, 1.26)	
**Has emotional/psychological support:**	*p = 0*.*023*	*p = 0*.*043*
No	1	1
Yes	0.81 (0.67, 0.97)	0.82 (0.67, 0.99)
**Social-economic group member:**	* p = 0*.*72*	
No	1	
Yes	0.97 (0.80, 1.16)	
**Ever used illicit drug:**	*p = 0*.*87*	
No	1	
Yes	1.03 (0.75, 1.40)	
**Locality:**	*p = 0*.*33*	*p = 0*.*55*
Rural	1	1
Urban	1.08 (0.92, 1.28)	1.07 (0.85, 1.36)
**HIV risks:**	*p = 0*.*73*	*p = 0*.*87*
Low	1	1
High	1.03 (0.87, 1.22)	0.98 (0.78, 1.24)
^**a**^**HIV status:**	*p = 0*.*034 *	*p = 0*.*038*
Negative	1	1
Positive	1.54 (1.04, 2.29)	1.54 (1.03, 2.31)
^*^Experienced violence from sexual partner:	* p <* .*001*	*p <* .*001*
No	1	1
Yes	1.68 (1.40, 2.01)	1.63 (1.36, 1.96)
^*^Engaged sex work	*p = 0*.*003*	*p = 0*.*023*
No	1	1
Yes	1.41 (1.12, 1.77)	1.31 (1.04, 1.65)

^**a**^12 observations missing

^*^ Analysis was restricted to 2276 sexually active AGYW and characteristics were measured in the last six months.

In a multivariate logistic regression analysis; having either incomplete or complete secondary school education (AOR = 1.36, 95% CI: 1.07–1.74), living with husband (AOR = 1.23, 95% CI: 1.02–1.47), HIV positive serostatus (AOR = 1.54, 95% CI: 1.03–2.31), having experienced violence from a sexual partner in the last six months (AOR = 1.63, 95% CI: 1.36–1.96), and having engaged in sex work in the last six months (AOR = 1.31, 95% CI: 1.04–1.65) and remained significantly associated with anxiety symptoms. Furthermore, AGYW who reported to have someone to depend on for emotional support were significantly less likely to screen positive for anxiety (AOR = 0.82, 95%CI: 0.67–0.99) (Table 2).

### Predictors of depression symptoms (PHQ-2 ≥ 3)

In an unadjusted analysis; age, marital status, education status, family composition, having children, having savings, HIV status and having experienced violence from a sexual partner in the last six months were associated with reporting symptoms of depression among AGYW (Table 3).

**Table 3:** Logistic regression analysis for depression and its predictors among AGYW.

	Depression (PHQ-2 ≥ 3)	
	*p*-value,	*p*-value,
	UOR (95% CI)	AOR (95%CI)
**Age (Years)**	*p <* .*001*	*p = 0*.*003*
15–19	1	1
20–23	1.55 (1.33, 1.81)	1.33 (1.10, 1.61)
**Marital status:**	*p = 0*.*002*	*p = 0*.*036*
Single	1	1
Currently-married	1.33 (1.14, 1.55)	1.59 (1.10, 2.30)
Formerly-married	1.15 (0.84, 1.59)	0.98 (0.68, 1.42)
**Education status:**	*p = 0*.*002*	*p = 0*.*001*
No formal/Incomplete primary education	1	1
Complete primary school	1.05 (0.88, 1.26)	1.10 (0.92, 1.32)
Complete/In complete secondary school	1.54 (1.24, 1.92)	1.55(1.22, 1.97)
**Family composition:**	*p <* .*001*	*p = 0*.*001*
Parents/relative or elder siblings	1	1
Husband	1.29 (1.11, 1.50)	0.83 (0.59, 1.17)
Alone/younger siblings/other	2.98 (1.79, 4.98)	2.51 (1.47, 4.29)
**Has children:**	* p = 0*.*001*	*p = 0*.*68*
No	1	*1*
Yes	1.28 (1.10, 1.50)	0.95 (0.76, 1.19)
**Has savings:**	*p = 0*.*004*	*p = 0*.*009*
No	1	1
Yes	0.80 (0.69, 0.93)	0.81 (0.70, 0.95)
**Has micro business:**	*p = 0*.*28*	
No	1	
Yes	1.09 (0.94, 1.26)	
**Physical deprivation:**	*p = 0*.*41*	
No	1	
Yes	0.92 (0.75, 1.12)	
**Has emotional/psychological support:**	*p = 0*.*06*	
No	1	
Yes	0.84 (0.70, 1.00)	
**Social-economic group member:**	* p = 0*.*72*	
No	1	
Yes	0.97 (0.81, 1.16)	
**Ever used illicit drug:**	*p = 0*.*10*	
No	1	
Yes	0.77 (0.57, 1.05)	
**Locality:**	*p = 0*.*25*	*p = 0*.*54*
Rural	*1*	1
Urban	*1*.*10 (0*.*94*, *1*.*28)*	1.08 (0.85, 1.35)
**HIV risks:**	*p = 0*.*51*	*p = 0*.*93*
Low	*1*	1
High	*1*.*06 (0*.*90*, *1*.*24)*	1.01 (0.80, 1.27)
^**a**^**HIV status:**	* p = 0*.*045*	*p = 0*.*081*
Negative	1	1
Positive	1.50 (1.01, 2.20)	1.43 (0.96, 2.14)
^*^Experienced violence from sexual partner:	*p <* .*001*	* p <* .*001*
No	1	1
Yes	1.92 (1.61, 2.29)	1.90 (1.59, 2.27)
^*^Engaged sex work	*p = 0*.*28*	
No	1	
Yes	1.13 (0.91, 1.42)	

^**a**^12observations missing

^*^ Analysis was restricted to 2276 sexually active AGYW and characteristics were measured in the last six months

In the multivariate logistic regression model; being aged 20 to 23 years (AOR = 1.33, 95% CI: 1.10–1.61), currently married (AOR = 1.59, 95% CI: 1.10–2.30), and having either incomplete or complete secondary school education (AOR = 1.55, 95% CI: 1.22–1.97) were significantly associated with depressive symptoms. Moreover, living alone, with younger siblings or others (AOR = 2.51, 95% CI: 1.47–4.290), and having experienced violence from sexual partners in the last six months (AOR = 1.90, 95% CI: 1.59–2.27) had a significant association with depressive symptoms in participants. Further, study participants who reported to have savings were significantly less likely to screen positive for depression (AOR = 0.81, 95%CI: 0.70–0.95) (Table 3).

### Predictors of anxiety and depression symptoms (PHQ-4)

In unadjusted POM analysis; age, marital status, education status, family composition, having children, having savings, HIV serostatus and having experienced violence from sexual partner in the last six months were associated with reporting severe and moderate symptoms of anxiety and depression symptoms compared to being in mild and normal categories (Table 4).

**Table 4:** Proportional odds model for the association between anxiety and depression and their predictors.

	*p-value*,	*p*-value,	*p-value*^†^
	UOR (95% CI)	AOR (95%CI)	* *
**Age (Years)**	*p <* .*001*	*P = 0*.*023*	
15–19	1	1	
20–23	1.41 (1.24, 1.61)	1.20 (1.03, 1.40)	0.67
**Marital status**	*p = 0*.*007*	*p = 0*.*42*	*0*.*06*
Single	1	1	*0*.*69*
Currently-married	1.24 (1.08, 1.42)	1.27 (0.88,1.82)	
Formerly-married	1.23 (0.93, 1.62)	1.08 (0.77,1.50)	
**Education status**	*p <* .*001*	*p <* .*001*	
No formal/Incomplete primary education	1	1	
Complete primary school	0.99 (0.85, 1.15)	1.03 (0.86, 1.23)	*0*.*49*
Complete/In complete secondary school	1.58 (1.30, 1.91)	1.60 (1.31, 1.97)	*0*.*35*
**Family composition**	*p <* .*001*	*p <* .*001*	
Parents/relative or elder siblings	1	1	
Husband	1.23 (1.08, 1.41)	0.98 (0.68, 1.41)	*0*.*24*
Alone/younger siblings/other	2.25 (1.42, 3.55)	1.89 (1.36, 2.62)	*0*.*65*
**Has children:**	*p = 0*.*003*	*p = 0*.*93*	
No	1	1	* *
Yes	1.23 (1.08, 1.41)	0.99 (0.83,1.19)	*0*.*8 *
**Has savings**	*p = 0*.*030*	*p = 0*.*07*	
No	1	1	
Yes	0.87 (0.76, 0.99)	0.87 (0.75, 1.01)	*0*.*14*
**Has micro business**	*p = 0*.*91*		
No	1		
Yes	1.01 (0.88, 1.15)		
**Physical deprivation**	*p = 0*.*81*		
No	1		
Yes	0.98 (0.82, 1.17)		
**Social-economic group member**	*p = 0*.*75*		
No	1		
Yes	0.98 (0.83, 1.14)		
**Ever used illicit drug**	*p = 0*.*23*		
No	1		
Yes	0.85 (0.65, 1.11)		
**Locality:**	*p = 0*.*09*	*p = 0*.*57*	
Rural	1	1	
Urban	1.13 (0.98, 1.29)	1.06 (0.86, 1.31)	0.18
**HIV risks:**	*p = 0*.*14*	*p = 0*.*64*	
Low	1	1	
High	1.11 (0.97, 1.28)	1.05 (0.86, 1.28)	0.91
^**a**^**HIV status**	*p = 0*.*006*	*p = 0*.*009*	
Negative	1	1	
Positive	1.65 (1.16, 2.34)	1.60 (1.13, 2.29)	*0*.*29*
^*^Experienced violence from sexual partner	p < .001		
No	1	1	
Yes	2.12 (1.81, 2.48)	2.12 (1.76, 2.55)	0.89
^*^Engaged sex work			
No	P = 0.09		
Yes	1.19 (0.98, 1.45)		

^**a**^12observations missing

^*^ Analysis was restricted to 2276 sexually active AGYW and characteristics were measured in the last six months;

^**†**^is a p-value from Brant test (testing POM assumption), overall was p = 0.35.

In the adjusted POM for ordinal multivariate logistic regression for anxiety and depression; participants aged 20–23 years (AOR = 1.20, 95% CI: 1.03–1.40), who have incomplete or complete secondary school education (AOR = 1.60, 95% CI: 1.31–1.97), and living alone or with younger siblings or others (AOR = 1.89, 95%CI: 1.36–2.62)were significantly associated with having severe and moderate symptoms of anxiety and depression compared to being in mild and normal categories of anxiety and depression. Furthermore, participants who were living with HIV (AOR = 1.60, 95% CI: 1.13–2.29), and those who reported having experienced violence from sexual partners in the last six months (AOR = 2.12, 95%CI: 1.76–2.55) were significantly more likely to screen positive for severe and moderate anxiety and depression compared to being in mild and normal categories(Table 4).

## Discussion

The current study adds to the knowledge base on the prevalence and predictors of anxiety and depressive symptoms among AGYW who are out of school in Tanzania. Findings from our study reveal that the prevalence of depressive (PHQ-2 ≥ 3) and anxiety (GAD-2 ≥ 3) symptoms among out-of-school AGYW were high, at 36% and 31% respectively. Furthermore, about 26% of the participants in the current study had at least moderate symptoms of anxiety and depression (PHQ-4 ≥ 6), suggesting that these individuals may have clinical depression and anxiety [43]. There is a paucity of literature on anxiety and depression among out of school AGYW. Therefore, comparisons were made with other studies involving both in and out of school populations. Although the age group of participants was slightly different(15–19 years) and majority were in school, prevalence of depression were similar to that seen among adolescents in Baltimore, USA (31%), Ibadan, Nigeria (29%) and lower than in Johannesburg, South Africa (45%)[44]. Another study which included both in and out of school AGYW found that 4% in Kenya and 9% in Zambia had moderate to severe symptoms of anxiety and depression [45]. The difference in findings between our study and the study in Kenya and Zambia may be because the current study only included AGYW who are out of school. A qualitative study conducted in Malawi suggested that AGYW who were out of school had limited social capital and struggled more to meet the basic needs compared to their school-attending peers [18], with potential resultant mental health symptoms [18,46].

The impact of depressive and anxiety disorders among AGYW is far-reaching and vast. This includes compromising the developmental potential of AGYW with resultant sub-optimal performance in social life, economic activities, and suicidality [29,47–49]. Both depression and anxiety are also associated with increased risk to HIV infection on-communicable diseases such as coronary heart diseases and diabetes [50,51]. Even worse, depression and anxiety are both associated with poor adherence to treatment, including oral glycemic control drugs and antiretroviral therapy (ART) [50]. For this reason, the World Health Organization (WHO) recommends routine screening and management of mental health disorders such as depression and anxiety as part of the essential package of HIV-related services for the key and vulnerable populations (KVP) such as AGYW[52]. And consequently, the Tanzania national guideline for the comprehensive package of HIV interventions for KVP also does the same [53]. However, similar to other low and middle-income countries [54], mental health services in Tanzania are mostly provided in tertiary facilities, limiting accessibility for the majority of the population including AGYW. It is therefore imperative that mental health services be expanded and strengthened to realize the attainment of the global health targets [55,56] and the achievement of the overall health of AGYW.

The finding on the association between secondary level of education and anxiety and/or depression is consistent with results from studies conducted in Mozambique [34] and Vietnam [57]. While causative analysis is lacking, it is possible that the association between education and depression is mediated through unemployment and lack of income-generating opportunities[58]. Coming from disadvantaged backgrounds, having secondary education but no employment may have led to anxiety and depression symptoms among AGYW[59]. Literature suggests that in-school, parenting and economic empowerment programs may help as primary prevention for common mental health disorders through improved cognition, self-esteem, capital and occupational status [60]. These may be adopted by mental health programs for primary prevention of depression and anxiety.

The findings regarding the association between marital status and depressive symptoms were conflicting. Our results show that AGYW who were currently married were significantly more likely to report depressive symptoms than those who were single. This finding is contrary to findings from a systematic review of studies in Ethiopia, which showed that the women who were divorced and widowed were more likely to report depressive symptoms than those who were single [61]. It is possible that masculinity entrenched within the culture of people within the current study region cultivates exploitative relationships within marital unions of AGYW with resultant depressive symptoms. A study in Uganda showed that mental health symptoms among women may be caused by poverty, unsupportive partners, food scarcity and intimate partner violence [62]. Further research may be needed to fully understand the factors which lead to depression and anxiety among AGYW as related to marriage or partnership status.

Findings from our study also underscore the contribution of family composition and sexual relationships to depressive and anxiety symptoms among AGYW who were out-of-school. In the current study, those who reported living with their husbands were more likely to report symptoms of anxiety compared to those who were living with their parents, relatives or elder siblings. Furthermore, those who were living alone, with younger siblings or others were more likely to report symptoms of depression and/or anxiety. It is possible that the stress linked to catering for their own needs or the needs of their younger sibling(s) in the context of limited resources cultivates this association. A study conducted in Mozambique showed a significant association between women who were heads of their households and depressive symptoms [34].

Anxiety symptoms among those living with their husbands may also be brought about by the high levels of violence experienced from sexual partners. Indeed, the current study shows over one-third of sexually-active AGYW reported having experienced violence from sexual partners in the last six months. Studies conducted across East and Southern Africa consistently document the association between violence from sexual partners and mental health symptoms [45,63–66]. Literature shows that women’s economic dependence on partners may also perpetuate partner violence[67–69], which may foster mental health symptoms among AGYW. Policies that ensure gender equality, women empowerment and partner violence protection have the potential to promote mental health among AGYW[60].

Although this was a small group, AGYW in the current study who were living with HIV were more likely to report symptoms of anxiety and depression. This finding is consistent with another study conducted among adolescents in Tanzania which showed an increased odds of depression among those living with HIV infection[24]. A study conducted in South Africa also shows that AGYW living with HIV experience more depressive symptoms compared to older women [70]. This association may be mediated by HIV related stigma, substance use as well as violence experience from partners among AGYW [70,71]. It is thus important to strengthen mental health and partner violence screening and prevention interventions within HIV programming. These factors have the potential to sustain HIV transmission through increased sexual risk behaviors and suboptimal adherence to ART [50,71].

The current study showed that having engaged in sex work in the previous six months was associated with an increased likelihood of reporting anxiety symptoms. Evidence shows that the sex work environment is associated with physical hostility and bullying from older female sex workers as well as harassment and other forms of violence from both the clients as well as community and local authorities[72–74]. These threats create a constant state of tension among AGYW who sell sex[74]. Even so, these adverse experiences are often unreported due to fear of negative repercussions especially in settings where sex work is criminalized[74] something that may further exacerbate mental health symptoms among AGYW who sell sex.

The findings of the current study also confirm the protective effect of economic status and social capital on depressive and anxiety symptoms. Having a person to rely on for emotional support was protective against anxiety and having savings protective against depressive symptoms. This implies that programs that help AGYW build social, networking as well as entrepreneur skills may help prevent anxiety and depression[60]. However, little evidence available is conflicting. While programs such as those offering conditional or unconditional cash transfers may promote mental health[60], others such as microcredit projects may lead to increased stress among participants [75]. Further research is needed to elucidate the impact of cash transfers and economic empowerment on depression and anxiety symptoms among AGYW.

### Implications for policy and practice

High prevalence of anxiety and depression among out of school AGYW calls for attention to mental health prevention, care, and treatment in this group. However, literature shows a lack of policies and implementation plans for mental health among AGYW in most low and middle-income countries [76,77]. Even where national mental health policies exist, there are no implementation plans in place to support the policy realization at local levels [77]. These issues coupled with the inadequate involvement of key stakeholders in policy development [77] may lead to suboptimal service provision to most-at-risk groups such as AGYW [78,79]. Strengthening mental health services should, therefore, begin with policy creation through the involvement of key stakeholders in order to form plans which respond to the needs of specific populations [78][79]. Implementation plans and guidelines should also be put in place to enable policy execution.

The link between mental health symptoms and HIV status, sex work underscores the need to strengthen the integration of routine mental health screening in HIV programming in order to enhance the health outcomes of AGYW [52,79]. This can be achieved through advocacy, decentralization of services, task-shifting and on-the-job training [54,79,80]. The primary prevention programs may leverage the association between mental health symptoms and violence, family composition, economic status as well as education level to advocate for social protection and other interventions that improve gender equality and reduce income inequality [79].

This study has some strengths; the large community sample of AGYW included improves the generalizability of our findings. The statistical analysis applied in our study considered the intrinsic ordering nature of anxiety and depression score outcome of the PHQ-4 screener, unlike using binary logistic regression analysis which could lead to loss of information and a decreased statistical power due to arbitrary dichotomization of anxiety and depression score outcome. However, the study also had some limitations: Firstly, data on the duration and nature of out of school status, and participants coming from the same households were not collected. Thus, these factors have not been adjusted for in our analysis. However, village which accounts for the highest level of clustering, age and education which are proxies for the nature of out of school status have been adjusted for in the analysis. Secondly, the PHQ-4 tool was not previously validated in Tanzania. In addition, the validation conducted in other settings reported a relatively lower sensitivity compared to structured clinical interviews [29–31]. This could have underestimated the prevalence in our study. However, being a very short tool, the PHQ-4 may be in busy primary health centers or community screening where a large number of people are attended [29,43]. This will enable a quick identification of those who most probably have anxiety and depression who can then be referred to higher-level facilities for diagnosis and management [29]. Thirdly, self-reported data on mental health and other variables have the potential for social desirability and recall biases, which would have made validation of the tool desirable. To offset this, we pre-tested and piloted the questionnaire with the embedded screener and made some adjustments following the pre-testing and pilot, and used the ACASI format of data collection, which is thought to be particularly helpful in sensitive questions since it reduces bias in discussing with an interviewer [81,82]. Fourthly, from the cross-sectional nature of the data, we cannot assess the causal relationship in the associations observed. Additionally, we did not conduct a qualitative assessment which would have helped answer some of the mechanisms by which the outcome variables were influenced. We recommend that future studies consider these improvements.

## Conclusion

In the study sample of out of school AGYW in Shinyanga, Tanzania, depressive and anxiety symptoms were prevalent, affecting over a third of the study population. These mental health disorder symptoms were associated with education level, family composition, violence from sexual partners, engaging in sex work as well as HIV status. Continued advocacy on mental health may help create awareness on primary preventive interventions including the creation of policies that enhance economic empowerment and gender equality. Scaling-up of mental health services including screening, diagnosis, and management among AGYW is also crucial for secondary prevention. These will help allow AGYW to achieve their full life potential.

## Supporting information

S1 Questionnaire (DOCX)

S1 Minimal data set (XLSX)
