# Supplementary material for: Prevalence and correlates of depression and anxiety symptoms among out-of-school adolescent girls and young women in Tanzania: A cross-sectional study
Source: PLoS One. 2019 Aug 16;14(8):e0221053. doi: 10.1371/journal.pone.0221053 (PMC6697336; doi:10.1371/journal.pone.0221053)
Supplement: S1 Questionnaire — (DOCX) [file pone.0221053.s001.docx]

**Questionnaire**

Participant ID: ____________________

**ACASI comprehension questions**

1. Are you in Tanzania now?
   1. Yes
   2. No
2. Are you a boy or a girl?
   1. Boy
   2. Girl

**Baseline questionnaire**

1. When were you born? |__|__|-|__|__|-|__|__|__|__|
2. What is your education level?
3. I have no formal education
4. Incomplete primary school education
5. Completed primary school education
6. Incomplete secondary school education
7. Completed secondary school education
8. Marital status
9. Single
10. Married (monogamous marriage)
11. Married (Polygamous marriage)
12. Cohabiting
13. Divorced/Separated
14. Widow
15. Who do you live with?
16. I live with my parents
17. I live with my relatives/friends
18. I live with my elder siblings
19. I live with my younger siblings
20. I live with my husband
21. I live alone
22. Other; specify______
23. Is your household receiving support from the Tanzania Social Action Fund (TASAF)?
24. Yes
25. No
26. In the last four weeks, have you ever stayed or slept hungry due to lack of food?
27. Yes
28. No
29. Do you have any person in your household or community, whom you can go to when in need advice or psychological support,?
    1. Yes
    2. No
30. Do you have children?
    1. Yes
    2. No
31. As of today, do you have savings?
    1. Yes
    2. No
32. Do you have any kind of business?
    1. Yes
    2. No
33. Are you currently a member of a loans and saving group?
    1. Yes
    2. No
34. Have you ever tried any of the following substances: *(check all that apply)*
35. Alcohol
36. Cigarettes
37. Cannabis
38. Heroin
39. Cocaine
40. Sniffing glue
41. Other; specify______
42. None
43. In the last six months, has any of your partner ever said or done anything to humiliate you in front of others? Threaten to hurt you or someone you care about? Insult you or make you feel bad about yourself?
    1. Yes
    2. No
44. In the last six months, has any of your partner ever used his hands /an object to hurt you physically? This might include; pushing you, shake or throwing something at you, slap you, punch you with a fist or with something that could hurt you, kick you, drag or beat you up, choke you or burn you on purpose or threaten to attack you with a knife, gun or weapon?
    1. Yes
    2. No
45. In the last six months, has any of your sexual partners ever: forced to have sex against your will? Have sex even when you did not want because of fear of what your partner would do? Or forced to do something sexually that you felt degrading or humiliating
    1. Yes
    2. No
46. In the last 6 months, have you ever negotiated with a man to be paid some money in exchange for sex?
    1. Yes
    2. No
47. Patient Health Questionnaire-4 (PHQ-4)

**Over the last 2 weeks, how often have you been bothered by the following problems?**

- 1. Feeling nervous, anxious or on edge

0. Not at all

1. Several days

2. More than half the days

3. Nearly every day

2. Not being able to stop or control worrying

0. Not at all

1. Several days

2. More than half the days

3. Nearly every day

3. Little interest or pleasure in doing things

0. Not at all

1. Several days

2. More than half the days

3. Nearly every day

4. Feeling down, depressed, or hopeless

0. Not at all

1. Several days

2. More than half the days

3. Nearly every day

1. HIV testing results
   1. Negative
   2. Positive
